# Prognostic Value and Immunological Role of KIFC1 in Hepatocellular Carcinoma

**DOI:** 10.3389/fmolb.2021.799651

**Published:** 2022-01-17

**Authors:** Dan Li, Tao Yu, Jingjing Han, Xu Xu, Jie Wu, Wei Song, Gang Liu, Hua Zhu, Zhi Zeng

**Affiliations:** ^1^ Department of Pharmacy, Renmin Hospital of Wuhan University, Wuhan, China; ^2^ Department of Oncology, Integrated Traditional Chinese and Western Medicine, The Central Hospital of Wuhan, Tongji Medical College, Huazhong University of Science and Technology, Wuhan, China; ^3^ Department of Infection Control, Renmin Hospital of Wuhan University, Wuhan, China; ^4^ Department of Geriatrics, Renmin Hospital of Wuhan University, Wuhan, China; ^5^ Department of Neurosurgery, Renmin Hospital of Wuhan University, Wuhan, China; ^6^ Department of Pathology, Renmin Hospital of Wuhan University, Wuhan, China

**Keywords:** KIFC1, liver hepatocellular carcinoma, prognosis biomarker, immune infiltration, ICB, immunotherapy

## Abstract

As one of the members of the kinesin family, the role and potential mechanism of kinesin family member C1 (KIFC1) in the development of liver hepatocellular carcinoma (LIHC), especially in the immune infiltration, have not been fully elucidated. In this study, multiple databases and immunohistochemistry were employed to analyze the role and molecular mechanism including the immune infiltration of KIFC1 in LIHC. Generally, KIFC1 mRNA expression was overexpressed in LIHC tissues than normal tissues, and its protein was also highly expressed in the LIHC. KIFC1 mRNA expression was correlated with tumor grade and TNM staging, which was negatively correlated with overall survival and disease-free survival. Moreover, univariable and multivariate Cox analysis revealed that upregulated KIFC1 mRNA is an independent prognostic factor for LIHC. The KIFC1 promoter methylation level was negatively associated with KIFC1 mRNA expression and advanced stages and grade in LIHC. The different methylation sites of KIFC1 had a different effect on the prognosis of LIHC. Specifically, the KIFC1 mRNA expression level showed intense correlation with tumor immunity, such as tumor-infiltrating immune cells and immune scores as well as multiple immune-related genes. Moreover, KIFC1 co-expressed with some immune checkpoints and related to the responses to immune checkpoint blockade (ICB) and chemotherapies. Significant GO analysis showed that genes correlated with KIFC1 served as catalytic activity, acting on DNA, tubulin binding, histone binding, ATPase activity, and protein serine/threonine kinase activity. KEGG pathway analysis showed that these genes related to KIFC1 are mainly enriched in signal pathways such as cell cycle, spliceosome, pyrimidine metabolism, and RNA transport. Conclusively, KIFC1 was upregulated and displayed a prognostic value in LIHC. Moreover, KIFC1 may be involved in the LIHC progression partially through immune evasion and serve as a predictor of ICB therapies and chemotherapies.

## Introduction

Primary liver cancer is one of the top 10 lethal tumors worldwide. China accounts for 55% of new liver cancer cases and related deaths every year (Torre et al., 2012). Liver hepatocellular carcinoma (LIHC) accounts for approximately 90% of primary liver cancer. An early diagnosis of LIHC is difficult because of its insipid onset and inconspicuous early symptoms. In clinical practice, patients often come to the hospital for treatment and diagnosis when they have symptoms in the late stage. At this time, the 5-year survival rate of patients with advanced LIHC is less than 5% due to the loss of active treatment opportunities or high recurrence and metastasis after treatment (Ziogas and Tsoulfas, 2017). At present, the development and clinical application of various targeted drugs for LIHC such as sorafenib extend the survival of patients to a certain degree (Hu et al., 2021). However, for another part of patients with advanced LIHC, targeted drugs did not show satisfactory efficacy. The total life expectancy is less than 1 year. Hence, it is necessary to develop novel and valuable biomarkers to help us for the accurate and early diagnosis of LIHC and find effective targets to further improve the therapeutic effect of liver cancer (Huang et al., 2017).

Studies have shown that abnormal expression of kinesin family genes plays a vital role in the occurrence and development of a variety of human cancers (Li et al., 2017; Li et al., 2020a). As a motor protein, it plays an important role in intracellular transport and cell division. Kinesin family member C1 (KIFC1) is a microtubule-dependent molecular kinesin with ATP activity and participates in a variety of cellular events, such as mitosis, meiosis, and macromolecular transport. A meta-analysis showed that high expression of KIFC1 can be used as a predictor for patients with non-small cell lung cancer, ovarian cancer, breast cancer, and LIHC. In addition, high levels of KIFC1 are associated with lymphatic metastasis (Sun et al., 2019). Therefore, KIFC1 may be a rational target for tumor therapy, which is worthy of further study. Previous studies have shown that KIFC1 can facilitate the occurrence of liver cancer. The possible mechanism is that TCF-4 activates KIFC1 and then upregulates the transcriptional activity of HMGA1 (Teng et al., 2019). KIFC1 is related to worse prognosis in patients with LIHC (Li et al., 2017), and the key mechanism is that KIFC1 not only can regulate the proliferation of HCC cells, but it can also reduce the apoptosis of HCC-LM3 and SMMC-7721 cell lines. Mechanistically, the apoptosis-related protein, B-cell lymphoma-2 (Bcl-2), was downregulated, whereas Bcl-2-associated X (Bax) and p53 protein were upregulated, and the expression levels of phosphorylated phosphoinositide 3-kinase (p-PI3K) and phosphorylated AKT were decreased significantly when KIFC1 was silenced (Fu et al., 2018). In addition, KIFC1 can promote invasion and metastasis through the signal pathway involving endothelial mesenchymal transition (Han et al., 2019). This process is carried out in a microtubule-dependent manner.

Although the incidence of cancer was increased partially by multiple genetic mutations, the impact of the tumor microenvironment (TME) on tumor progression or immune response did not attract enough attention (Li et al., 2019; Zhu et al., 2021). Recently, cytokine and immune checkpoint blockade (ICB) therapy have become treatment strategies for various types of cancers (Long et al., 2017; Zhang et al., 2021). As a consequence, biomarkers that predict response in immune and stromal cells may help determine which patients will benefit most from ICB treatment. Besides the reported mechanism, the potential mechanisms about KIFC1 in the TME involved in the formation and progression of LIHC have not been elucidated; few studies have addressed the relationship between KIFC1 expression and immune cell infiltration in LIHC. Whether KIFC1 can provide guidance for immunotherapy as well as chemotherapy in LIHC has not been studied.

Therefore, multiple bioinformatics methods and clinical samples were used to comprehensively evaluate the relationship between KIFC1 expression and clinicopathological features as well as the prognosis of LIHC. In addition, the relationship between KIFC1 gene promoter methylation and prognosis were analyzed. The correlation of KIFC1 with tumor immune cell infiltration, immune checkpoints, immune-related genes, and responses to ICB and drugs was detected. The results provide novel insights on the function of KIFC1 and new goals for the diagnosis and prognosis of LIHC.

## Methods

### HCCDB Database Analysis

The HCCDB database (http://lifeome.net/database/hccdb/home.html) includes the liver cancer gene expression profile data in GEO, TCGA-LIHC, and ICGC LIRI-JP (Lian et al., 2018). A total of 3,917 samples were divided into 15 data sets. We use HCCDB to analyze the KIFC1 expression difference between hepatocellular carcinoma tissues and normal liver tissues.

### GEO and TCGA Analysis

Four LIHC datasets were enrolled in this study including GSE14520, GSE57957, GSE36376, and TCGA. Among the GEO datasets, GSE14520 included 217 paired non-cancerous and LIHC samples, while there are 36 paired tumor and adjacent non-cancerous liver samples in GSE57957; in addition, there are 193 normal tissues and 240 LIHC tissues in GSE36376. Likewise, the TCGA database contained 159 normal liver tissues and 371 primary liver tumor samples. In this study, we used different datasets from multiple databases to investigate the KIFC1 expression difference to make sure that the results are authentic.

### Association Between DNA Methylation of KIFC1 and Prognosis of LIHC

The URL of the UALCAN database is http://ualcan.path.uab.edu, which can query and analyze the expression difference of genes between tumor and normal samples (Chandrashekar et al., 2017). We can also compare the expression differences and promoter methylation levels among different tumor subgroups and different clinical characteristics groups. In addition, it can also be used to analyze the impact of a gene on the prognosis of a tumor. We used this database to analyze KIFC1 expression and its promoter methylation levels in normal and cancerous tissues according to the individual cancer stage and grade of patients. The results are shown in the box chart. The association between the KIFC1 methylation value and expression level was assessed by the MethHC (http://methhc.mbc.nctu.edu.tw/php/index.php) tool. The MethSurv data set (https://biit.cs.ut.ee/methsurv/) was used to assess the DNA methylation and the association between KIFC1 methylation and the prognosis of LIHC.

### Exploring the Association of Survival With KIFC1 in LIHC

We performed univariate and multivariate Cox regression analyses to determine the appropriate terms for building nomograms. A forest was applied to display the *p*-value, HR, and 95% CI for each variable by using the “forestplot” R package through R software. A nomogram was created according to the results of the multivariate Cox proportional hazards analysis to predict the total recurrence rate in 1, 3, and 5 years. The “rms” R package was used to evaluate the risk of recurrence for individual patients by the points associated with each risk factor.

We extracted survival information for each sample in TCGA. Indicators such as OS, disease-specific survival (DSS), disease-free survival (DFS), and progression-free survival (PFS) were used to elucidate the relationship between KIFC1 and the prognosis of LIHC patients. Kaplan–Meier (KM) and log-rank tests were used for the survival analysis of LIHC (*p* < 0.05), and survival curves were analyzed by the “survminer” and “survivor” R packages.

### GEPIA Analysis

Gene Expression Profiling Interactive Analysis (GEPIA) is a newly developed interactive web server. Its website is http://gepia.cancer-pku.cn/index.html. This database provides online analysis based on data from TCGA and GTeX projects (Tang et al., 2017). In our study, we used the given TCGA expression dataset to assess the difference of KIFC1 between LIHC and normal liver tissues and to confirm the results of gene expression analysis in the HCCDB database and GEO database. In addition, we used GEPIA to analyze the prognostic significance of KIFC1 mRNA expression in LIHC and draw the survival curve with log rank *p*-value.

### LinkedOmics Analysis

LinkedOmics is a public website on http://www.linkedomics.org/login.php, which contains 32 TCGA cancer types of multiple omics data, including federation generated from the Clinical Proteomics Tumor Analysis Consortium (Vasaikar et al., 2018). We applied this online tool to identify and analyze KIFC1 co-expression genes in hepatocellular carcinoma cohort from TCGA, which contains 371 samples. The data from Linkfinder are signed and sorted; after that, GO analyses, including molecular function (MF), biological process (BP), cellular component (CC), and KEGG analysis, are performed by the gene set enrichment analysis (GSEA) method.

### Association Between KIFC1 and Immune Infiltration, Immune-Related Genes, Immune Checkpoints, and Immune Checkpoint Blockade Responses

The TIMER database is an online tool that is used to evaluate the immune infiltration of various types of cancers to explore the immunological, clinical, and genomic characteristics of cancer. Its website is https://cistrome.shinyapps.io/timer (Li et al., 2020b). The ESTIMATE algorithm was applied to calculate immune and stromal scores for each sample (Yoshihara et al., 2013). In this study, we firstly investigated the expression of KIFC1 in different cancers using TIMER. The correlation between the expression of KIFC1 and the abundance of tumor-infiltrating immune cells and the corresponding cellular genetic markers was also analyzed. The results are presented in box diagrams and tables.

We further investigated the correlation of KIFC1 with immune cell infiltration by the CIBERSORT algorithm using the R packages “ggplot2,” “ggpubr,” and “ggExtra.” In addition, co-expression analyses between KIFC1 and immune-related genes or immune checkpoints were performed using the R packages “limma,” “reshape2,” “RColorBrewer,” “ggplot2,” “pheatmap,” and “immuneeconv.” The TIDE algorithm was used to predict potential ICB responses (Zhu et al., 1950; Jiang et al., 2018).

### The Human Protein Atlas Project

The Human Protein Atlas Project (HPAP) (https://www.proteinatlas.org/) is a dataset for analyzing the expression of different proteins. It contains 16,975 unique proteins from different kinds of human tissues and cells. We used it to evaluate the KIFC1 expression in protein levels in LIHC and normal liver tissues.

### Clinical Tissue Samples and Data Collection

A total of 36 patients with LIHC who did not receive radiotherapy or chemotherapy before surgery (26 men and 10 women; age range, 23–73 years) were included in the present study. The LIHC tissues and their matched non-cancerous liver tissues were formalin-fixed and paraffin-embedded to compare the KIFC1 protein expression difference for validation.

### Immunohistochemistry

IHC staining procedures were performed according to a standard protocol that was described in detail in our previous study (Zeng et al., 2020). The paraffin sections were placed in xylene for 15 min twice for dewaxing and rehydrated in gradient alcohol for 5 min. The sections were treated with 3% H_2_O_2_ and then antigen retrieval by a citric acid buffer (pH 6.0). Then, the sections were blocked with 5% bovine serum albumin for 20 min and incubated with a rabbit anti-human KIFC1 antibody solution (1:100; Proteintech, Manchester, United Kingdom) at 4°C overnight (16–18 h). Then sections were incubated with horseradish peroxidase-labeled polymer with secondary antibody [UltraSensitiveTM SP (Mouse/Rabbit) IHC Kit-9710; Maixin Bio, Fuzhou, China] at room temperature for 15 min each. After washing with PBS for three times, the reaction products were stained with 3,3′-diaminobenzidine and lightly counterstained with hematoxylin. The sections with PBS instead of the primary antibody served as negative control. The total staining score was used to evaluate the protein expression of KIFC1 in normal and tumor tissues. The total staining score is the multiplication of the score of staining intensity and staining range. In this study, regardless of staining intensity, the staining score was regarded as positive if the proportion of positive cells is greater than 5%.

### Correlation of KIFC1 With Drug Sensitivity

We collected LIHC-related RNA sequences and clinicopathological and survival data while keeping samples with recorded clinical information from TGCA. We predicted the chemotherapeutic response for samples of cancers that had a correlation of KIFC1 with OS according to the available pharmacogenomics database [the Genomics of Drug Sensitivity in Cancer (GDSC), https://www.cancerrxgene.org/]. The prediction of the half-maximum inhibitory concentration (IC50) of the samples was achieved by ridge regression and the prediction accuracy through R package “pRRophetic.” All parameters were set by the default values with the removal of the batch effect of the “combat” and tissue type of “allSoldTumours,” and duplicate gene expression was summarized as the mean value.

### Statistical Analysis

Statistical analyses were conducted using R software (version 4.0.2). The analysis results of HCCDB database showed the *p*-value. The analysis of KIFC1 expression in TIMER and GEO databases showed *p*-value. The survival curve generated by GEPIA analysis showed *p*-value. The difference of KIFC1 and its methylation levels in different clinical feature groups was analyzed by the UALCAN database, and the correlation of the prognosis of LIHC with KIFC1 methylation was analyzed by the MethSurv database and the *p*-value was given. T-test was used to estimate the significance of gene expression level between groups. Univariate and multivariate Cox regression analyses were conducted to analyze the effect of KIFC1 on the prognosis of LIHC. Forest was used to show the *p*-value, HR, and 95% CI of each variable. Spearman correlation analysis was used for genetic correlation, and *p* < 0.05 was considered statistically significant.

## Results

### Expression of KIFC1 in LIHC

**FIGURE 1:**
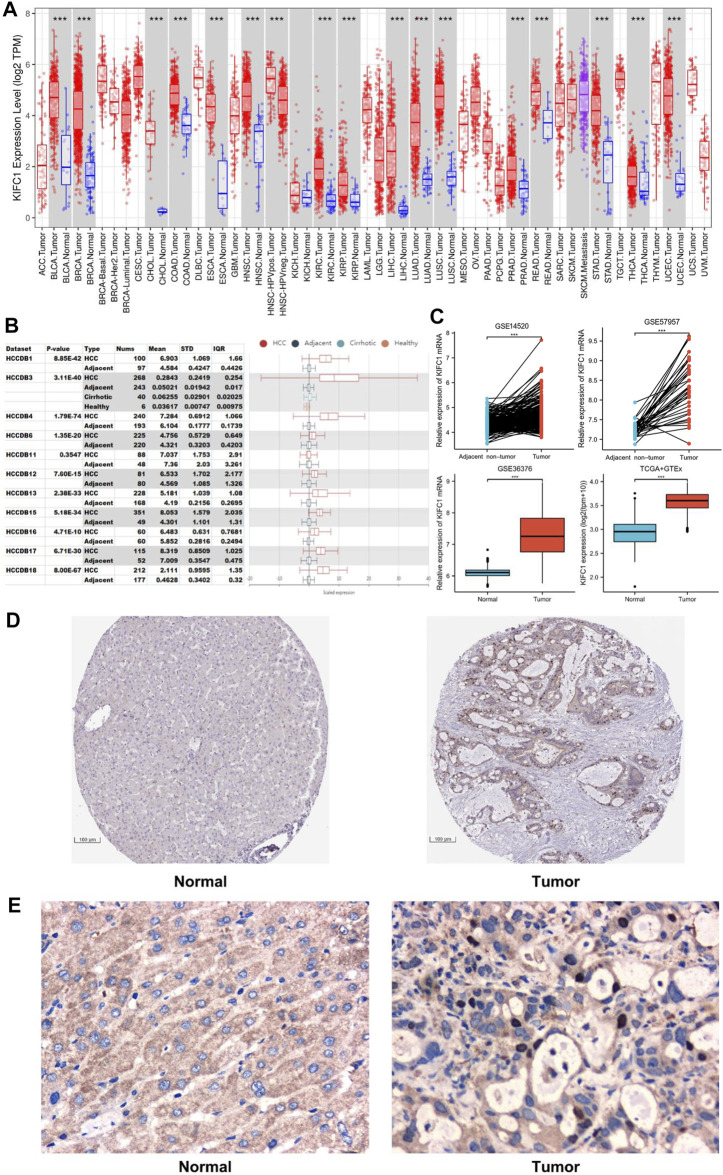
The expression level of KIFC1 in LIHC from different databases. **(A)** KIFC1 expression levels in different tumor types in TIMER. **(B)** KIFC1 expression in tumor tissues and the adjacent normal tissues in HCCDB. **(C)** KIFC1 mRNA expression difference in LIHC and adjacent non-cancerous or normal liver tissues from GSE14520, GSE57957, GSE36376. and TCGA. **(D)** IHC profile of KIFC1 protein expression in normal and LIHC tissues from HPA database. **(E)** Typical IHC staining of KIFC1 protein expression in normal and LIHC tissues from clinical samples. The *p*-value was calculated using Student’s t-test. *p < 0.05, **p < 0.01, ***p < 0.001.

Firstly, in order to more accurately understand the expression of KIFC1 between LIHC and normal liver tissue, we used three different kinds of databases for confirmation. The data from TCGA were analyzed using the TIMER online tool. The results indicated that the mRNA expression of KIFC1 in LIHC was higher than in normal tissues (*p* < 0.001) (Figure 1A). Secondly, 11 LIHC cohorts from the specialized liver cancer database HCCDB were analyzed, and the same trends were obtained (Figure 1B). Thirdly, from analyzing the KIFC1 mRNA expression from GSE14520, GSE57957, GSE36376, and TCGA, it indicated that the KIFC1 mRNA expression was markedly higher in LIHC compared with its adjacent non-cancerous tissues or normal liver tissues (*p* < 0.001) (Figure 1C). Besides, the IHC profile from HPA database showed a higher expression in LIHC tissues (Figure 1D). Moreover, the protein expression analysis from our clinical samples with IHC also indicated that KIFC1 was located and highly expressed near the nucleus of liver cancer cells, which was statistically different from that in normal liver tissues (8/36 vs. 1/36, chi-square test, *p* < 0.05) (Supplementary Figure S1). However, no significant difference was observed in cytoplasm (Figure 1E).

### The Expression of KIFC1 Is Associated With Patients’ Survival in LIHC

**FIGURE 2:**
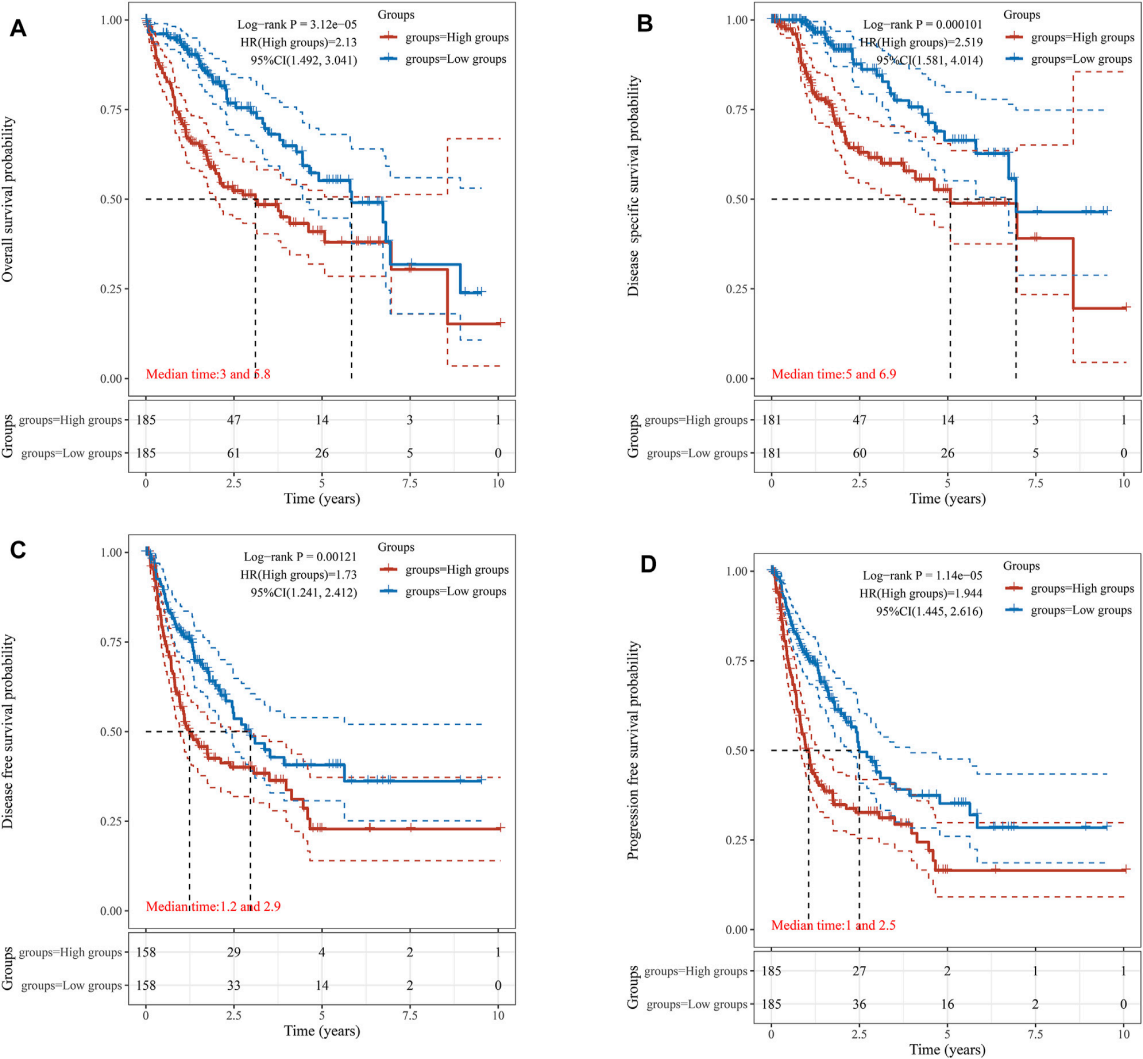
Association of KIFC1 expression with patient survival curve. **(A)** Overall survival curve. **(B)** Disease-specific survival. **(C)** Disease-free survival. **(D)** Progression-free survival.

The survival data of KIFC1 expression in LIHC patients were analyzed. Kaplan–Meier survival curves were used to evaluate the relationship between the KIFC1 expression and survival outcomes. The cut-off value of the high and low KIFC1 expression group was set as the median. The results showed that patients with higher KIFC1 mRNA expression had shorter OS (*p* = 3.12e-05) (Figure 2A), DSS (*p* = 0.00001) (Figure 2B), DFS (*p* = 0.00121) (Figure 2C), and PFS (*p* = 1.14e-05) (Figure 2D). Consequently, the expression of KIFC1 mRNA in LIHC is associated with survival. The result from GEPIA is also consistent with our results (Supplementary Figure S2).

### Prognostic Potential of KIFC1 in LIHC

**FIGURE 3:**
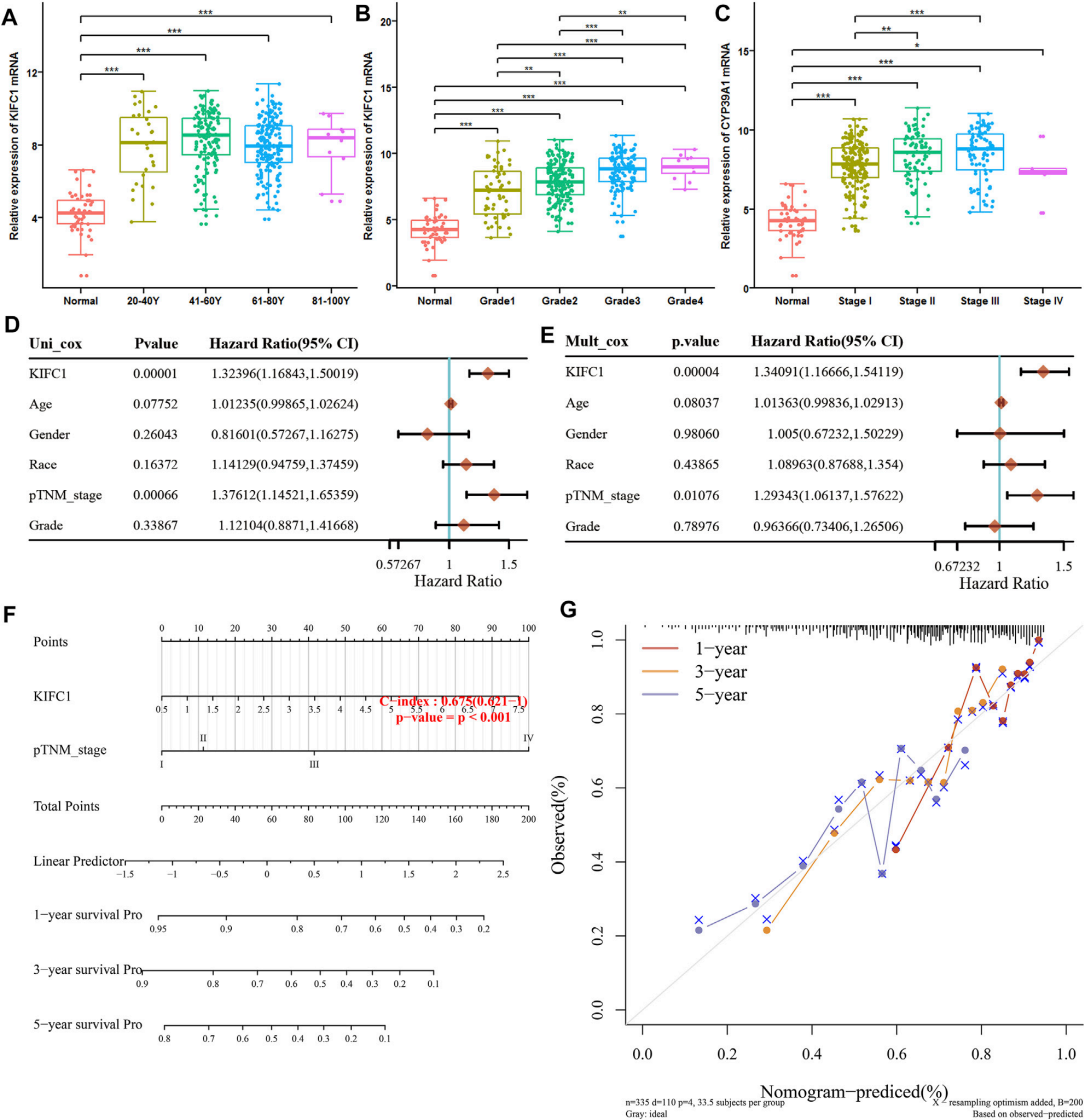
Univariate and multivariate analysis of prognostic factors of LIHC. **(A)** Relative expression of KIFC1 in subgroups of patients with LIHC based on age. **(B)** Relative expression of KIFC1 in subgroups of patients with LIHC based on grade. **(C)** Expression of KIFC1 in subgroups of LIHC patients based on stage. **(D, E)** Hazard ratio and *p*-value of constituents involved in univariate and multivariate Cox regression of the KIFC1 gene and other clinical characteristics. **(F)** Nomogram predicting the 1-year, 3-years and 5-year OS of LIHC patients. **(G)** The calibration plots for predicting patient 1-year, 3-year, and 5-year OS. *p < 0.05, **p < 0.01, ***p < 0.001.

For further subgroup analysis, the correlation of KIFC1 mRNA expression with multiple clinicopathological features was performed in the UALCAN database, which includes 371 LIHC samples. It revealed that the transcription level of KIFC1 was markedly higher compared with normal liver tissue in different subgroups according to age, tumor stage, and grade (*p* < 0.001). Moreover, KIFC1 expression was positively related to the first three stages and grades of LIHC (Figures 3A–C), excluding stage IV and grade IV. Furthermore, univariate and multivariate Cox regression analyses illustrated that KIFC1 expression (*p* < 0.001) and pTNM-stage (*p* < 0.05) were important independent factors to the prognosis of LIHC (Figures 3D,E). We further constructed a nomogram that combined only two independent prognostic factors (including KIFC1 and pTNM-stage) to provide a quantitative guideline for clinicians to predict the probability of 1-, 3-, and 5-year OS in LIHC patients (Figure 3F). Each patient is given a total score through adding each prognostic parameter point, with a higher total score meaning a worse outcome for that patient. In addition, the calibration curves showed that the nomogram performed well in estimating 1-, 3-, and 5-year OS (Figure 3G). Therefore, KIFC1 may be a potential diagnostic marker for LIHC.

### Relationship Between KIFC1 Methylation and KIFC1 Expression, Prognosis of LIHC

**FIGURE 4:**
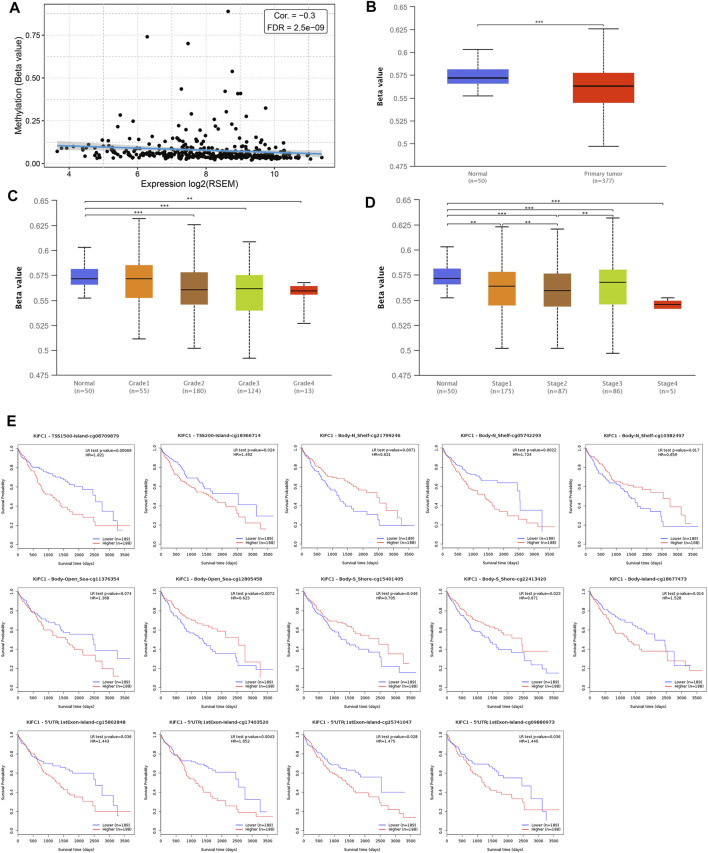
Association of KIFC1 methylation level and its expression in LIHC. **(A)** Correlation between KIFC1 methylation and its expression level. **(B)** The promoter methylation level of KIFC1 in LIHC and normal tissues. **(C, D)** The correlation between promoter methylation level of KIHC1 with different grade and tumor stages. **(E)** The impact of different methylation sites of KIFC1 on OS of LIHC patients analyzed by MethSurv webtool. The *p*-value was calculated using Student’s t-test. *p < 0.05, **p < 0.01, ***p < 0.001.

DNA methylation significantly affects gene expression in cancer. MethHC analysis showed that the KIFC1 mRNA was negatively correlated with its promoter methylation level (*r* = −0.3, FDR = 2.5e-09) (Figure 4A). Moreover, the methylation level of the KIFC1 promoter in liver cancer is downregulated than normal and was negatively correlated with the individual cancer grade and stage of the LIHC patient (Figures 4B–D). In addition, using the MethSurv tool, we found that the LIHC patients with high methylation values of cg08709879 were associated with poor OS (*p* = 0.00069) (Figure 4E). The other methylation sites that had a significant association with the prognosis of LIHC were also presented in Figure 4E.

### Relationship Between KIFC1 and Immune Cell Infiltration in LIHC

**FIGURE 5:**
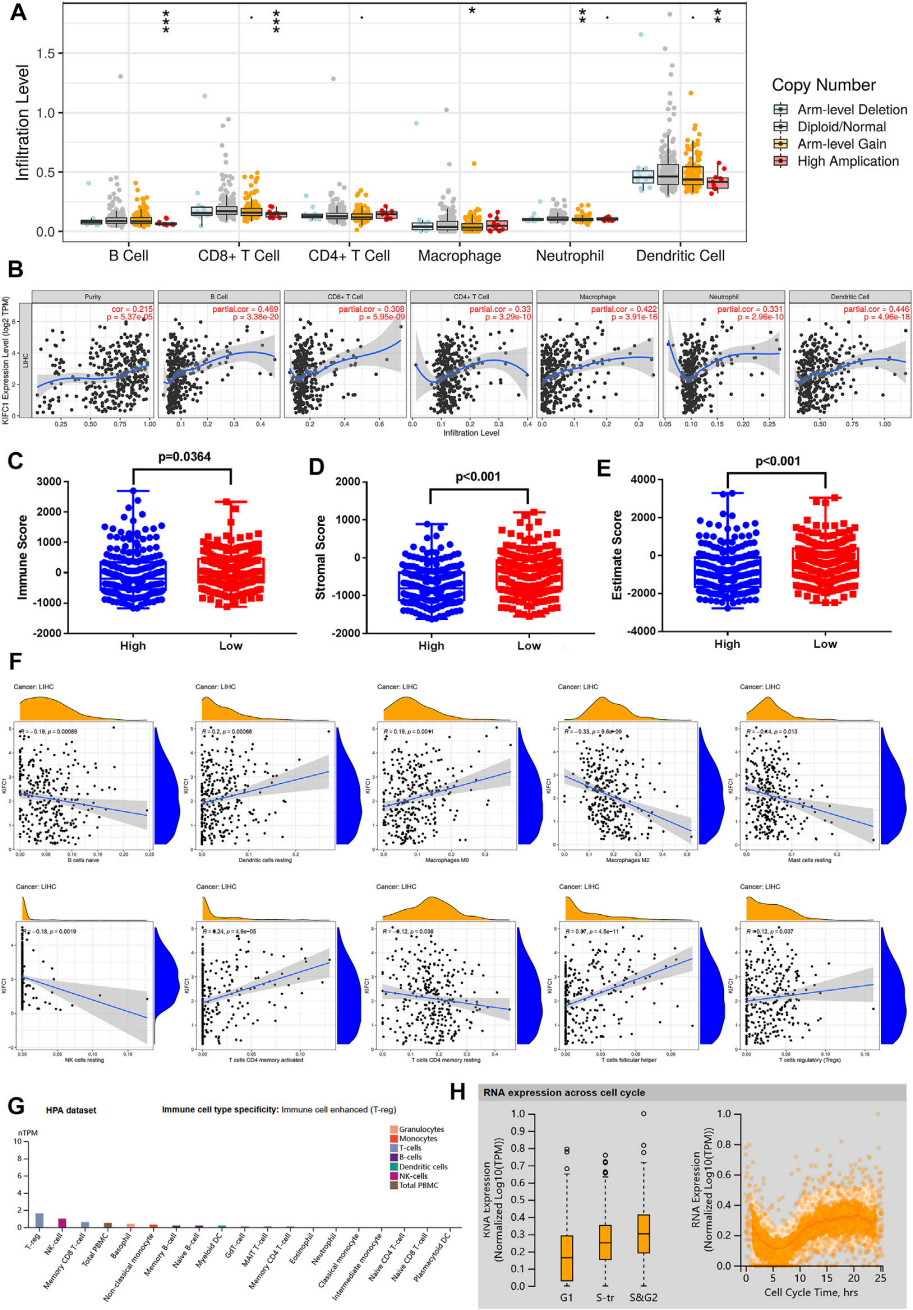
KIFC1 expression was associated with immune infiltration level in LIHC. **(A)** The immune infiltration cells in LIHC. **(B)** KIFC1 expression level is related to the degree of immune infiltration in LIHC from TIMER. Correlation of KIFC1 with immune **(C)**, stromal **(D)**, and estimate **(E)** scores. **(F)** Association between LIFC1 and immune infiltration levels analyzed by CIBERSORT algorithm. The *p*-value was calculated using Student’s t-test. *p < 0.05, **p < 0.01, ***p < 0.001. **(G)** Different mRNA expression levels of KIFC1 in immune cells. **(H)** Association between KIFC1 expression and cell cycle.

To further explore how the KIFC1 gene affects tumor progression, TIMER was employed to analyze the correlation of KIFC1 expression with the level of immune invasion. The results indicated that KIFC1 copy number variation was related to the infiltration levels of B cells, CD8+ T cells, macrophages, neutrophils, and dendritic cells (Figure 5A). Specifically, the expression of KIFC1 was notably associated with tumor purity (*r* = 0.215, *p* = 5.37e-05) and the level of major infiltrating immune cell as B cells, CD8+ T cells, CD4+ T cells, macrophages, neutrophils, and dendritic cells (Figure 5B). In order to evaluate whether KIFC1 expression will affect the tumor immunity, the patients were divided into high and low KIFC1 expression groups, and the immune score was calculated; results indicated that KIFC1 expression was associated with immune and stromal scores (Figures 5C–E). We further used the CIBERSORT algorithm to investigate the tumor-infiltrating immune cells, we found that the expression of KIFC1 was negatively correlated with the infiltration of naïve B cells, M2 macrophages, resting mast cells, resting natural killer (NK) cells, and resting memory CD4+ T cells (Figure 5F). However, the results indicated that KIFC1 expression was positively associated with the infiltration of resting dendritic cells, M0 macrophages, follicular helper T cells, activated memory CD4+ T cells, and regulatory T cells (Figure 5F). In addition, we discovered that in immune cells, KIFC1 was highly expressed in Tregs (Figure 5G). The variation in transcript expression was correlated to the cell cycle. The KIFC1 RNA expression peak phase was G2 (Figure 5H), indicating that KIFC1 had a role in the cell cycle profile. This suggests that KIFC1 plays a critical role in regulating immune cell infiltration in LIHC.

### Correlation Analysis Between KIFC1 Expression and Immune Cell Markers, Immune-Related Genes

In order to further confirm the correlation of KIFC1 with immune-infiltrating cells, we focus next on the relationship between KIFC1 and various immune cell marker genes. In addition to the six types of immune cells as shown in Figure 5, it is worth mentioning that T cells with various functions are also included, such as Th1 cells, Th2 cells, Tfh cells, Th17 cells, Tregs, and depleted T cells. It indicated that KIFC1 expression was notably related to the immune markers of most immune T cells in LIHC, especially with Th1, Th2, Treg, and T-cell exhaustion markers (Table 1). Interestingly, we found no correlation between KIFC1 and the M2 macrophage marker and Th17 marker in LIHC. Thus, we could further confirm the specific association with immune infiltrating cells in the LIHC microenvironment.

**TABLE 1:** Correlation between KIFC1 and immune cell markers for LIHC.

Description	Gene markers	Cor	*p*-Value	*P* Star
CD8+ T cell	CD8A	0.203398	8.22E-05	**
	CD8B	0.200194	0.000103	**
T cell (general)	CD3D	0.262876	3.09E-07	**
	CD3E	0.175419	0.000701	**
B cell	CD19	0.286148	2.01E-08	**
	CD79A	0.154896	0.002776	**
Monocyte	CD86	0.265267	2.40E-07	**
	CD115 (CSF1R)	0.132829	0.010477	*
TAM	CCL2	0.039289	0.450371	
	CD68	0.197832	0.000129	**
M1				
Macrophage	INOS (NOS2)	−0.0387	0.457349	
	IRF5	0.434875	0	**
	COX2 (PTGS2)	0.041373	0.426875	
M2				
Macrophage	CD163	0.04255	0.413647	
	VSIG4	0.053858	0.300694	
	MS4A4A	0.050356	0.333247	
	CD66b			
Neutrophils	(CEACAM8)	0.154884	0.002778	**
	CD11b (ITGAM)	0.252297	9.28E-07	**
	CCR7	0.043555	0.402704	
Natural killer cell	KIR2DL1	−0.00925	0.859044	
	KIR2DL3	0.122901	0.017873	*
	KIR2DL4	0.215475	2.84E-05	**
	KIR3DL1	0.050247	0.334464	
	KIR3DL2	0.171911	0.000885	**
	KIR3DL3	0.094199	0.069939	
	KIR2DS4	0.044908	0.388409	
Dendritic cell	HLA-DPB1	0.169081	0.001094	**
	HLA-DQB1	0.162709	0.001684	**
	HLA-DRA	0.152419	0.003278	**
	HLA-DPA1	0.112032	0.03102	*
	BDCA-1 (CD1C)	0.118305	0.022664	*
	BDCA-4 (NRP1)	0.200391	0.000105	**
	CD11c (ITGAX)	0.279898	4.78E-08	**
Th1	T-bet (TBX21)	0.063403	0.222987	
	STAT4	0.233002	6.11E-06	**
	STAT1	0.364553	5.76E-13	**
	IFN-γ (IFNG)	0.275613	6.83E-08	**
	TNF-α (TNF)	0.222424	1.53E-05	**
Th2	GATA3	0.152103	0.003344	**
	STAT6	0.132692	0.010557	*
	STAT5A	0.274166	9.09E-08	**
	IL13	0.121788	0.018944	*
Tfh	BCL6	0.169588	0.001056	**
	IL21	0.192633	0.000189	**
Th17	STAT3	0.087206	0.093487	
	IL17A	0.084484	0.104228	
Treg	FOXP3	0.137818	0.007895	**
	CCR8	0.339772	1.77E-11	**
	STAT5B	0.302215	3.41E-09	**
	TGFβ (TGFB1)	0.268872	1.62E-07	**
T-cell exhaustion	PD-1 (PDCD1)	0.329187	7.96E-11	**
	CTLA4	0.316216	4.64E-10	**
	LAG3	0.318865	4.10E-10	**
	TIM-3 (HAVCR2)	0.264626	2.57E-07	**
	GZMB	0.102434	0.048696	*

Cor, R-value of Spearman’s correlation; TAM, tumor-associated macrophage

*p < 0.05; **p < 0.01.

**FIGURE 6:**
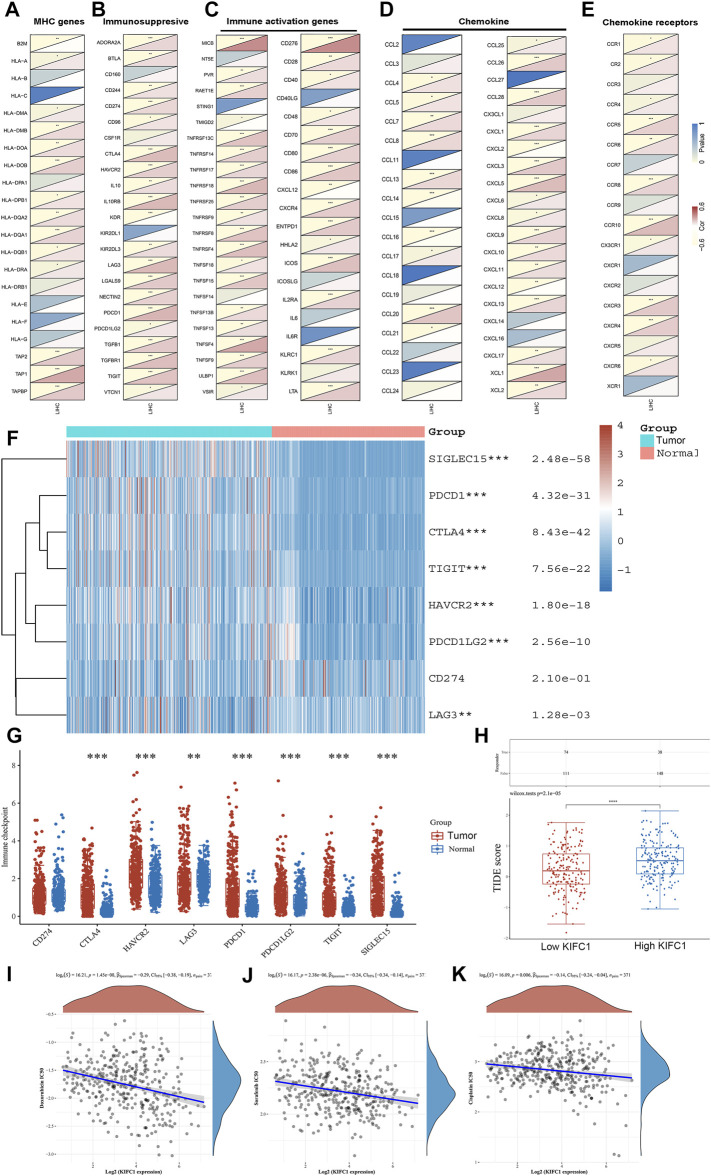
Co-expression of KIFC1 with **(A)** MHC, **(B)** immunosuppressive, **(C)** immune activation, **(D)** chemokine, and **(E)** chemokine receptor genes. **(F, G)** Different expressions of immune checkpoints in LIHC and normal patients. **(H)** Different responses to ICB therapy in low and high KIFC1 expressions. **(I–K)** Correlations between KIFC1 and the IC50 of chemotherapy drugs. *p < 0.05, **p < 0.01, ***p < 0.001.

We further assessed the co-expression of KIFC1 with MHC, immunosuppressive, immune activation, chemokine, and chemokine receptor genes. The results demonstrated that KIFC1 was co-expressed with several MHC genes, especially TAP1 (Figure 6A). KIFC1 was positively co-expressed with almost all immunosuppressive genes except KIR2DL1, KDR, CSF1R, and CD160 (Figure 6B). KIFC1 also had a significant correlation with immune activation genes, particularly MICB and CD276 (Figure 6C). For chemokine and chemokine receptor genes, KIFC1 had a high correlation with XCL1 and CCR10 (Figures 6D,E).

### Correlation of KIFC1 With the Sensitivity of ICB and Chemotherapies

We further predicted the association between KIFC1 expression and ICB response. Firstly, we evaluated the expression of immune checkpoints (including SIGLEC15, PDCD1, CTLA4, TIGIT, PDCD1LG2, CD274, HAVCR2, and LAG3) in LIHC and normal tissues. We found that these immune checkpoints, except CD274, were all highly expressed in LIHC (Figures 6F,G). Moreover, we found that KIFC1 was positively co-expressed with these immune checkpoints, except SIGLEC15 (Table 2). Additionally, LIHC patients with high KIFC1 expression had a high TIDE score, indicating that these patients may had a better response to ICB therapy (Figure 6H). We also assessed the relationship between KIFC1 and the chemotherapeutic drug that is usually used in LIHC, we found that KIFC1 was negatively related to the IC50 of doxorubicin, sorafenib, and cisplatin (Figures 6I–K). KIFC1 may be a predictor of ICB and chemotherapies.

**TABLE 2:** Correlation of KIFC1 with immune checkpoints in LIHC.

Genes	Cor	*p*-value
TIGIT	0.267	1.548e-07
CD274	0.171	0.0009
HAVCR2	0.217	2.408e-05
PDCD1LG2	0.116	0.024
SIGLEC15	0.070	0.175
LAG3	0.302	2.427e-09
CTLA4	0.296	5.405e-09
PDCD1	0.324	1.456e-10

### Enrichment Analysis of KIFC1 Neighborhood Genes in LIHC

**FIGURE 7:**
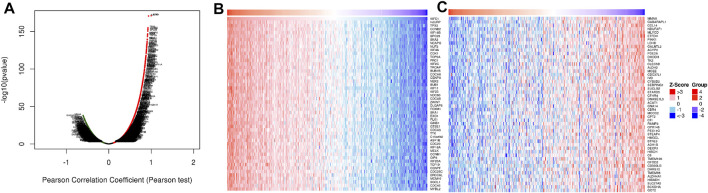
Genes differentially expressed in association with KIFC1 in LIHC. **(A)** Volcano plot. **(B, C)** Heat maps of the top 50 genes positively and negatively correlated with KIFC1.

**FIGURE 8:**
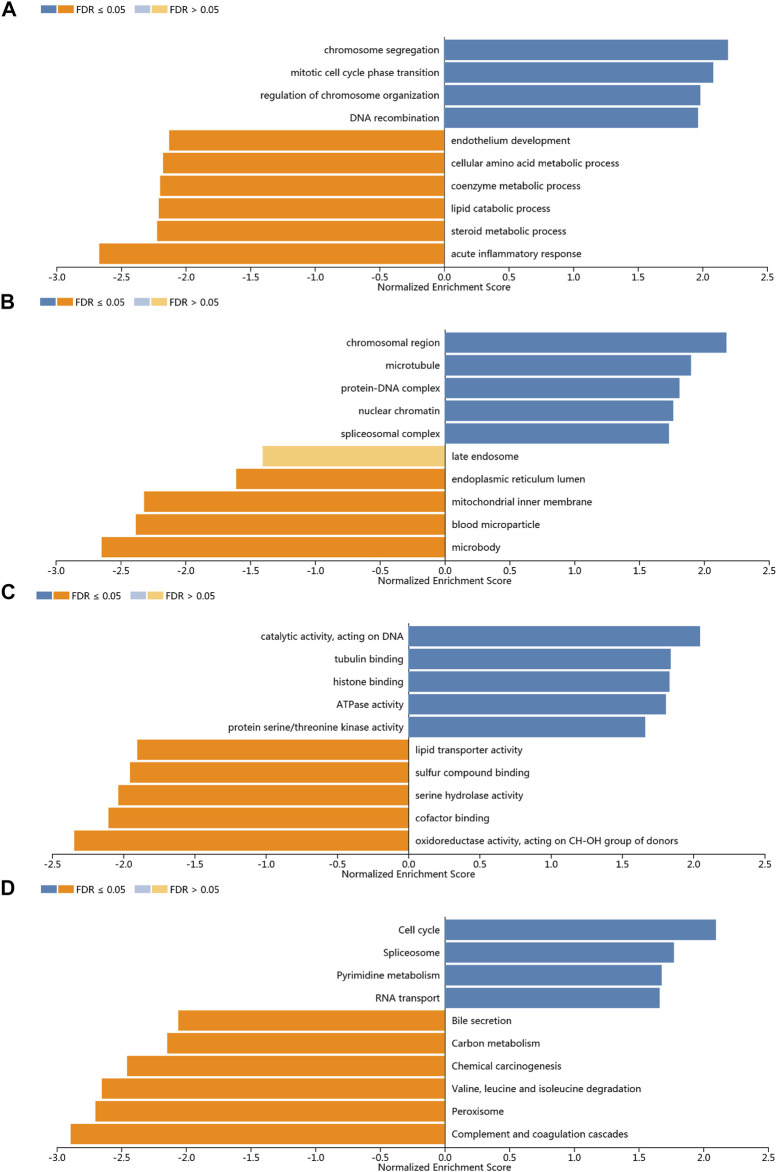
Enrichment analysis of the genes altered in the KIFC1 neighborhood in LIHC. **(A)** Biological processes. **(B)** Cellular components. **(C)** Molecular functions. **(D)** KEGG pathway analysis.

In order to study the functional network of KIFC1 neighborhood genes in LIHC, we first identified KIFC1 neighborhood genes with LinkedOmics. The results are shown in a volcanic plot (Figure 7A). In addition, the top 50 negatively and positively significant related genes were shown in the heat map, respectively (Figures 7B,C). Significant GO term analysis by GSEA indicated that genes correlated with KIFC1 were located mainly in the chromosomal region, microtubule, protein–DNA complex, nuclear chromatin, and spliceosome complex, where they were involved in chromosome segregation, mitotic cell cycle phase transition, the regulation of chromosome organization, and DNA recombination. These related genes also served as catalytic activity, acting on DNA, tubulin binding, histone binding, ATPase activity, and protein serine/threonine kinase activity (Figures 8A–C). KEGG pathway analysis demonstrated that these genes related to KIFC1 were mainly enriched in signal pathways such as cell cycle, spliceosome, pyrimidine metabolism, and RNA transport (Figure 8D).

## Discussion

Due to the characteristics of delayed diagnosis and high recurrence of liver cancer, there are still some patients in clinic with unsatisfactory treatment effect and poor prognosis; therefore, it is important to explore novel genes related to the occurrence and development of LIHC and find markers with higher specificity and sensitivity, especially the other molecular mechanism targets such as immune infiltration, to improve the therapy of LIHC. This study aims to explore the diagnostic value of KIFC1 gene in LIHC and its influence on tumor immune invasion. As a member of the kinesin superfamily, the main function of KIFC1 is to act as a motor protein affecting microtubule dynamics and function, including spindle formation and the regulation of centrosome amplification (Xiao et al., 2017). The overexpression of KIFC1 has been reported to lead to the formation of monopolar spindles in tumor cells. This promotes the process of the division and proliferation of tumor cells (Mittal et al., 2016). Several studies have verified that KIFC1 expression is related with poor prognosis in multiple tumors, such as non-small cell lung cancer, renal cell carcinoma, and breast cancer, and it does happen primarily by affecting the proliferation of tumor cells (Liu et al., 2016; Li et al., 2018). Taken together, we can infer that KIFC1 may be involved in tumor formation and development by further affecting cell proliferation through its effect on microtubules.

In our study, we found that KIFC1 was differentially expressed in various types of cancers compared with its corresponding normal tissues such as BLCA, BRCA, CHOL, COAD, ESCA, LIHC, LUAD, LUSC, PRAD, READ, STAD, THCA, UCEC, HNSC, KIRC, and KIRP. Then, KIFC1 expression was further validated in liver cancer, and it indicated that patients with higher KIFC1 expression had worse prognosis. An IHC analysis of tissues from the HPA database and clinical samples indicated that there was a higher expression of KIFC1 protein in LIHC tissues than in normal tissues. Additionally, we also found that KIFC1 gene expression was correlated with tumor stage and clinical grade. In addition, patients with high expression of KIFC1 have a worse prognosis. Furthermore, univariate and multivariate Cox regression analyses revealed that KIFC1 expression was an independent prognosis factor of LIHC. Therefore, KIFC1 has a potential diagnostic value in LIHC. Interestingly, in this study, KIFC1 protein was highly expressed in the nucleus of LIHC cells; however, there was no significant difference in the cytoplasm between LIHC and normal liver tissues. It was found in a previous study that KIFC1 plays a potential function in nuclear formation and the nuclear localization sequence on N-terminal domain, which is critical for the translocation of KIFC1 into the nucleus (Wei et al., 2019) as it reported that KIFC1 showed specific transport characteristics during the cell cycle, which is involved in regulating DNA synthesis in S phase and chromatin maintenance in mitosis (Wei and Yang, 2019). These all indicated that KIFC1 may play its function by transporting into the nucleus. In addition, it is consistent with a previous study that high expression of KIFC1 in the nucleus was correlated with worse OS, which could serve as an independent biomarker for African-American triple-negative breast cancer (Ogden et al., 2017). In our study, the differential expression of KIFC1 also mainly exists in the nucleus instead of cytoplasm, and the higher expression in the nucleus contributed to the progression of LIHC.

It has been reported that abnormal DNA molecular changes are the initiator of tumors, which appear in the early stage of tumors and accompany with the whole process of tumor development and are closely related to the prognosis of tumors (Cabel et al., 2017). DNA methylation, copy number variation, gene mutation, and microsatellite sequence changes are all important components of tumor epigenetics. However, DNA methylation is more prevalent in almost all tumors than the latter three, which can directly regulate gene expression (Schübeler, 2015). Therefore, the relationship between methylation and the prognosis of liver cancer can be explored. Abnormal methylated genes may serve as markers for the early diagnosis and prognostic evaluation of LIHC (Liu et al., 2020a). In this study, we used UALCAN to analyze the promoter methylation of the KIFC1 gene in liver cancer and its relationship with clinical characteristics. The results showed that KIFC1 was hypomethylated in LIHC tissues regardless of stage. Although KIFC1 methylation was not significantly different among different stages or grades, it showed a gradual decreasing trend with the increase of stages. This result partly explains that the increased expression of KIFC1 is regulated by methylation. Likewise, it was found in a previous study that targeted genes NEFH and SMPD3 were hypermethylated in paired LIHC samples than in the normal samples, which were lower expressed in LIHC samples; thereby, they could serve as tumor suppressor genes in LIHC (Revill et al., 2013). Similarly, it was reported that NAT1 mRNA was reduced in colorectal carcinoma compared with normal tissues, while the methylation of the promoter region of NAT1 was higher in colorectal carcinoma than that in normal tissues (Shi et al., 2019). We also found that the different methylation sites of the KIFC1 gene had a different effect on the prognosis of LIHC patients as shown in Figure 4E. These all suggested that DNA methylation affects the expression of targeted genes and then affects the tumor cell behavior.

Next, we analyzed the KIFC1-related pathway in LIHC to understand its carcinogenic mechanism. Based on GO and KEGG analyses, we observed that the functional network of KIFC1 in LIHC was related to the chromosome segregation, mitotic cell cycle phase transition, the regulation of chromosome organization, and DNA recombination. They also served as catalytic activity, acting on DNA, tubulin binding, histone binding, ATPase activity, and protein serine/threonine kinase activity. KEGG pathway analysis indicated that KIFC1-related genes were enriched in cell cycle, spliceosome, pyrimidine metabolism, and RNA transport. All these results suggested that KIFC1 regulates cell cycle by affecting mitosis, mainly by affecting chromatin tissue regulation and tubulin binding. It is consistent with previous studies that KIFC1 can affect microtubules (Ma et al., 2017).

To further evaluate the potential immune mechanisms of KIFC1 in LIHC, we secondly analyzed KIFC1-related immune infiltration levels. The results demonstrate that the KIFC1 expression level is strongly positive correlated with the infiltration level of B cells, CD8+ T cells, macrophages, neutrophils, and dendritic cells in LIHC. In addition, the expression of KIFC1 was negatively correlated with the infiltration of naïve B cells, M2 macrophages, resting mast cells, resting NK cells, and resting memory CD4+ T cells but positively correlated with the infiltration of resting dendritic cells, M0 macrophages, activated memory CD4+ T cells, follicular helper T cells, and regulatory T cells. After analyzing the correlation between KIFC1 expression and immune cell marker genes, the results revealed that the function of KIFC1 in regulating different immune infiltrating cells is different. Likewise, immune score evaluation indicated that KIFC1 expression was significantly correlated with immune and stromal score, which suggested that KIFC1 level was linked to the level of immune infiltration.

Studies have shown that T-cell infiltration in tumor tissue as a protective factor can inhibit tumor invasion and metastasis. Patients with higher levels of T-cell infiltration often have a better prognosis (Oh et al., 2020; van der Leun et al., 2020). In this study, there was a positive correlation between KIFC1 and T-cell marker genes (CD3D and CD3E). Hence, we hypothesized that patients with high KIFC1 expression may have a better prognosis, but the opposite was true. Firstly, it is known that tumor-associated macrophages (TAMs) are the most plentiful immune cells in TME. CD68 is the most reliable marker of macrophages, and IRF5 of M1 macrophages, both of which are positively correlated with KIFC1. This indicates that KIFC1 may play a part in regulating the polarization of TAM. Researchers have found that TAM can promote tumor cells’ growth and metastasis through a variety of pathways (Batoon and McCauley, 2021; Kong et al., 2021). This could partly explain why KIFC1 can promote LIHC progress. Secondly, it is well known that Treg cells can inhibit the function of T cells and are an important factor in maintaining the immune tolerance of the body. However, in tumors, Treg cells become accomplices of cancer cells to help them escape the immune surveillance of the body, leading to tumor progression and metastasis (Liu et al., 2020b). Moreover, in immune cells, the mRNA expression of LIFC1 was the highest in Tregs. FOXP3, CCR8, STAT5b, and TGFB1 are genetic markers of Treg cells. T-cell depletion is the main factor that causes the immune dysfunction in tumor patients, of which, tumor cells and TME can induce the expression of PD1 on activated T cells and activate relevant signaling pathways, leading to T-cell depletion (Wang et al., 2020). PD-1, CTLA4, LAG3, HAVCR2, and GZMB are T-cell depletion markers, and all of these marker genes are positively correlated with KIFC1 expression in LIHC in this study. Thus, the high expression of KIFC1 may be a factor involved in the T-cell depletion process. This is the second reason why KIFC1 promotes LIHC progression through the immune pathway. Most importantly, we investigated the correlation of KIFC1 and immune checkpoints, including SIGLEC15, TIGIT, CTLA4, CD274, HAVCR2, LAG3, PDCD1, and PDCD1LG2, which were associated with the response to ICB (Marwitz et al., 2017; Nebhan and Johnson, 2020). These immune checkpoints except CD274 were all highly expressed in LIHC. Moreover, we found that KIFC1 was positively co-expressed with these immune checkpoints, except SIGLEC15. As high expression of immune checkpoints was associated with T-cell exhaustion and worse prognosis, this also partly explained the cancer-promoting effect of KIFC1. Additionally, LIHC patients with high KIFC1 expression may had a better response to ICB therapy, indicating that LIHC patients with high KIFC1 expression were more suitable for ICB therapy. Interestingly, KIFC1 was negatively related to the IC50 of doxorubicin, sorafenib, and cisplatin. These results indicated that KIFC1 may be a predictor of ICB and chemotherapeutics; for example, LIHC patients with high KIFC1 expression may have a better response to ICB, doxorubicin, sorafenib, and cisplatin therapy. These findings provided new ideas for the precise treatment of LIHC patients.

Taken together, the effect of KIFC1 on immune cell infiltration has both positive and negative effects on tumor patients. The inhibitory and promotive effects on the tumor are coexisting. The negative side accounts for a greater proportion and therefore appears to promote tumor progression. The limitation is that we do not yet understand the mechanism involved in this process by which KIFC1 influences immune cell infiltration. However, it is clear that KIFC1 is involved in the recruitment and regulation of immune infiltrating cell in LIHC. In this study, our study differs from previous literature in that we found the differential expression of KIFC1 in the location of nucleus that contributes to the occurrence and development of LIHC, and hypomethylation may explain the higher expression of KIFC1 in LIHC. Most importantly, KIFC1 expression may affect the immune microenvironment and then indirectly affects the prognosis of LIHC and serves as a predictor of ICB therapies and chemotherapeutics. However, the limitation of this study is that a large number of samples were needed to verify our results, and more clinical samples will be collected to enrich the data in the future. Moreover, the underlying immune mechanisms should be explored and KIFC1 as biomarkers to predict the immune response rate in real-world LIHC patients should be conducted.

## Conclusion

Conclusively, the increased expression of KIFC1 in LIHC was associated with increased levels of different immune cells infiltration level with a worse prognosis. The lower level of promoter methylation may be the reason for the increased expression of the KIFC1 gene in LIHC cells. KIFC1 alters the clinical outcomes of patients with LIHC by affecting immune cells in the TME, and it may be used as an independent predictor of ICB therapies and chemotherapeutics.

## Data Availability

The datasets presented in this study can be found in online repositories. The names of the repository/repositories and accession number(s) can be found in the article/Supplementary Material.

## Supplementary Material

Supplementary Figure S1 Protein expression analysis from our clinical samples with IHC (8/36 vs. 1/36, chi-square test, *p* < 0.05).

Supplementary Figure S2 Association of KIFC1 expression with patient survival curve. **(A)** Overall survival curve. **(B)** Disease-free survival.
