# Supplementary figures and images for: Prognostic Value and Immunological Role of KIFC1 in Hepatocellular Carcinoma
Source: Front Mol Biosci. 2022 Jan 17;8:799651. doi: 10.3389/fmolb.2021.799651 (PMC8802309; doi:10.3389/fmolb.2021.799651)

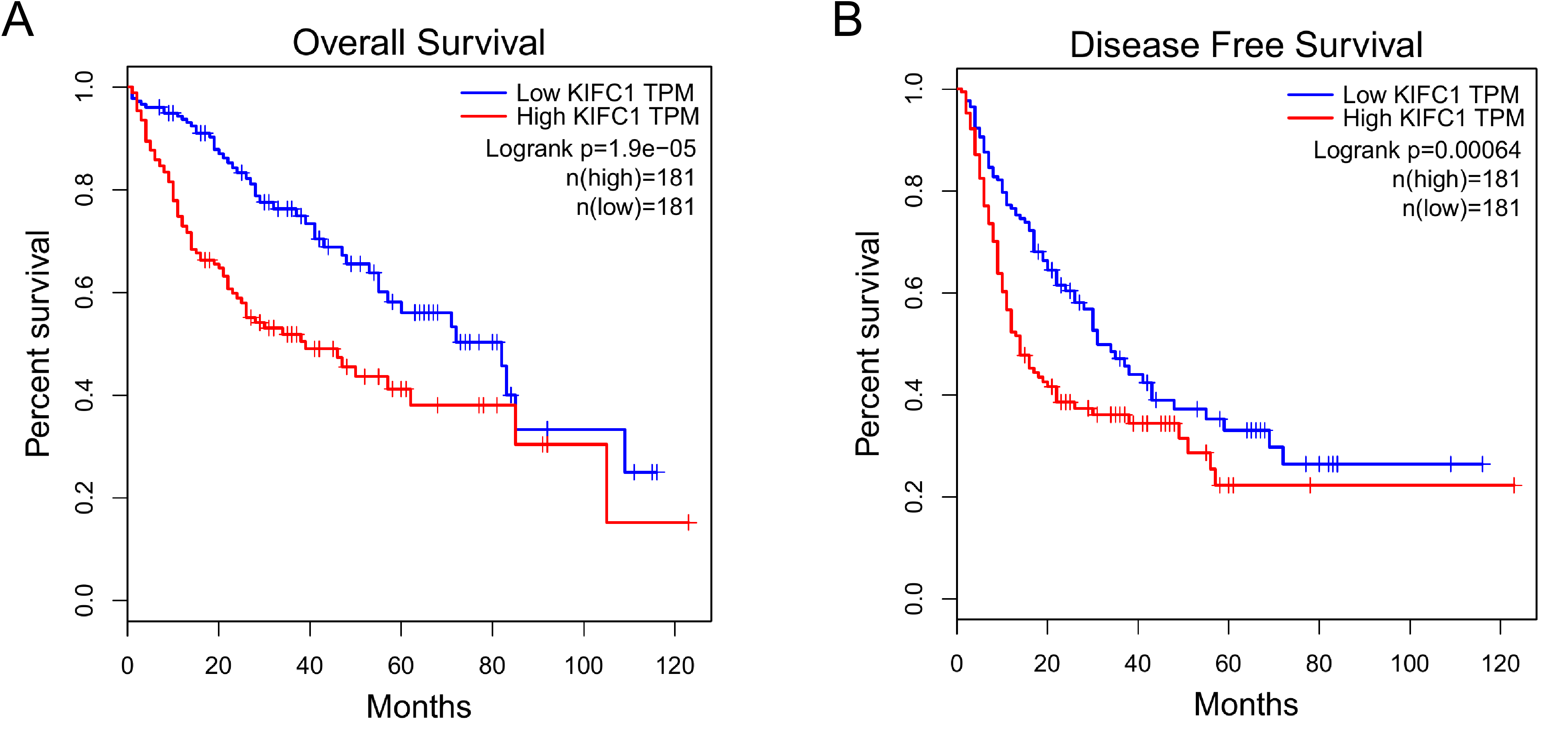

Supplement: Supplementary file 1 [file Image2.PNG]

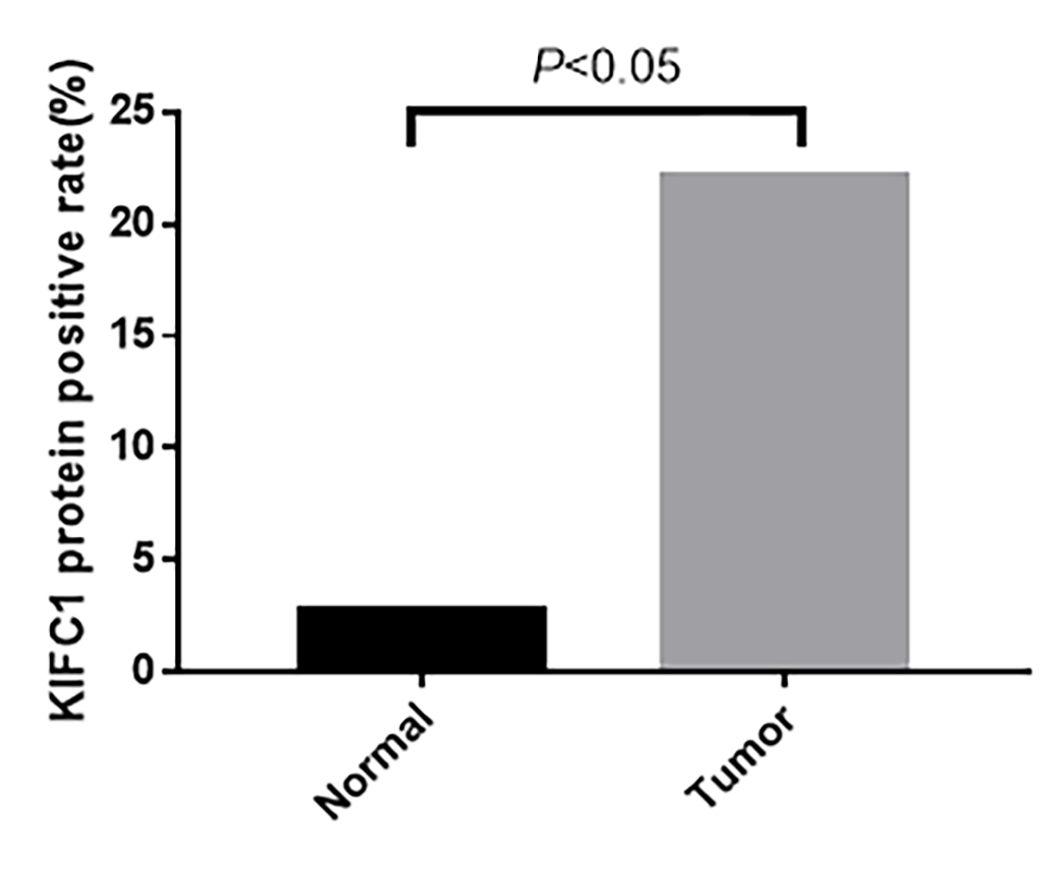

Supplement: Supplementary file 2 [file Image1.PNG]
